# Role of chest CT scan in patients with preexisting cancer and COVID-19 pneumonia

**DOI:** 10.1186/s12880-023-00984-w

**Published:** 2023-02-06

**Authors:** Faezeh Khorasanizadeh, Soori Kaviani, Shadi Salamroudi, Monireh Sadat Seyyedsalehi, Masoumeh Gity, Kazem Zendehdel

**Affiliations:** 1 Advanced Diagnostic and Interventional Radiology Research Center (ADIR), Tehran University of Medical Sciences, Tehran, Iran; 2 Department of Radiology, Tehran University of Medical Sciences, Tehran, Iran; 3 Cancer Research Centre, Cancer Institute, Tehran University of Medical Sciences, Tehran, Iran; 4 Department of Medical and Surgical Sciences, University of Bologna, Bologna, Italy

**Keywords:** COVID-19, Chest CT scan, Cancer, Atypical finding, Radiology

## Abstract

**Background:**

Detection of COVID-19 in cancer patients is challenging due to probable preexisting pulmonary infiltration caused by many infectious and non-infectious etiologies. We evaluated chest CT scan findings of COVID-19 pneumonia in cancer patients and explored its prognostic role in mortality.

**Methods:**

We studied 266 COVID-19 patients with a history of cancer diagnosis between 2020 and 2022. Chest CT images were reported based on Radiological Society of North America (RSNA) structural report and the CT score and pattern of involvement were noted. We used multivariate logistic regression models to determine the association between CT scan findings and mortality of the cancer COVID-19 patients.

**Results:**

The mean age was 56.48 (± 18.59), and 53% were men. Gastrointestinal (29.3%), hematologic (26.3%), and breast (10.5%) cancers were the most frequent types of cancer. The prevalence of atypical or indeterminate findings in the chest CT was 42.8%. Most radiologic findings were consolidation mixed with ground-glass opacity (44.4%), pleural effusion (33.5%), and pure ground-glass opacity (19.5%). The risk of death was higher among those who had typical chest CT for COVID-19 (OR 3.47; 95% CI 1.14–8.98) and those who had a severity of score higher than 18 (OR 1.89; 95% CI 1.07–3.34). Also, presence of consolidation (*P *value 0.040), pleural effusion (*P *value 0.000), centrilobular nodules (*P *value 0.013), and architectural distortion (*P *value 0.005) were associated with a poorer prognosis.

**Conclusion:**

Less than half of COVID-19 patients with a history of cancer had typical imaging features of COVID-19. Radiologists should be aware of atypical, rare, or subtle chest CT findings in patients with pre-existing cancer.

## Introduction

Cancer patients, with an annual incidence of more than 18 million new cases, may account for a sizable portion of the COVID-19 infected population [1]. They are more susceptible to infection, with higher hospitalization rates and severe outcomes than the general population. Several reports identify potential prognostic factors for severe illness and higher mortality, such as increased age, male sex, smoking status, number of comorbidities, and poorer performance status [2, 3]. However, evidence on the prognostic role of cancer type, active cancer, anticancer therapy type, recent surgery, and metastatic stage of cancer in published articles is still controversial and heterogeneous [4].

Due to the limited availability and sensitivity of viral testing kits during the pandemic, chest CT has been considered a critical component for evaluating patients with suspected or known COVID-19 infection [5]. Cancer patients may have underlying lung disease due to various etiologies. Some reasons may include primary lung cancer, pulmonary metastasis, opportunistic infections such as fungal disease, atypical viral bronchopneumonia due to an immunocompromised state, chemotherapy-induced pulmonary changes, and pulmonary reaction to radiotherapy. As a result, distinguishing between the various causes of radiologic pulmonary infiltration in these patients is not always straightforward and may pose diagnostic challenges. Data from the normal populations demonstrated the role of CT scans in predicting mortality rates and revealed higher CT scores. The most common CT findings were ground glass opacities and consolidation with bilateral and peripheral distribution, but less common features could also be identified[6]. Several CT features are associated with poor prognosis, including crazy-paving patterns, multilobar involvement, consolidations, architectural distortion, and traction bronchiectasis [5, 7–9]. However, to ourknowledge, no sufficient studies have been published to explore the effects of underlying cancer on the imaging appearance of COVID-19 and its prognostic role in mortality in this specific group [10–13].

This study aims to evaluate COVID-19 imaging findings in patients with preexisting cancer and its prognostic role as an independent factor in determining disease outcomes in the oncologic setting.

## Materials and methods

### Study population

Our study used data from 266 COVID-19 patients with a cancer history registered in Imam Khomeini Hospital’s Clinical COVID-19 Registry between 2020 and 2022. Clinical evaluation, including CT scan or RT-PCR test results, confirmed COVID-19 infection. Cancer patients who had a chest CT scan within five days of the onset of their symptoms were included in the study. The COVID-19 registry collected demographic and clinical information about COVID-19 and cancer disease, including oxygen saturation, length of hospital stay, ICU hospitalization, intubation and mortality rates, type of cancer, stage of cancer, oncologic treatment, and the time between cancer diagnosis and the last time receiving anti-tumor treatment. Because of the small sample size, cancer was classified as hematological, lung, breast, GI, and other tumors.

### CT scan protocol

CT scan systems (SOMATOM Emotion 16 scanner; Siemens) were used to obtain non-enhanced chest CT images in the supine position. CT scan images were acquired during a single inspiratory breath-hold to minimize motion artifacts. We used tube voltage = 80–110 kVp, effective current 60–80 mA, pitch = 1–1.5, matrix = 512 × 512, slice thickness = 5 mm (reconstructed slice thickness = 1.5 mm), and pulmonary U90S kernel to minimize patient radiation exposure. The reconstructed images were uploaded to the picture archiving and communication system (PACS). The Iranian Society of Radiology COVID-19 Consultant Group (ISRCC) recommended the low-dose CT scan protocol, which did not cause any issues with image interpretation [14].

### Chest CT image interpretation

Two radiologists reviewed all chest CT scans concurrently with both lung (width, 1500 HU; level, − 700 HU) and mediastinal (width, 350 HU; level, 40 HU) windows. Following the final agreement, the prepared checklist was completed. Both radiologists were blinded to the patient’s information and outcome during the review. The CT scan was evaluated in four areas: morphology, CT scan involvement score, associated pulmonary lesions, and mediastinal findings. Morphology features included pure ground-glass opacity (GGO), consolidation, predominant GGO, predominant consolidation, and other associated pulmonary abnormalities, including crazy-paving pattern (a combination of GGO with superimposed interlobular and intralobular septal thickening), pleural effusion, pericardial effusion, lymphadenopathy, centrilobular nodules, architectural distortion, metastatic nodules, and mass.

**Fig. 1:**
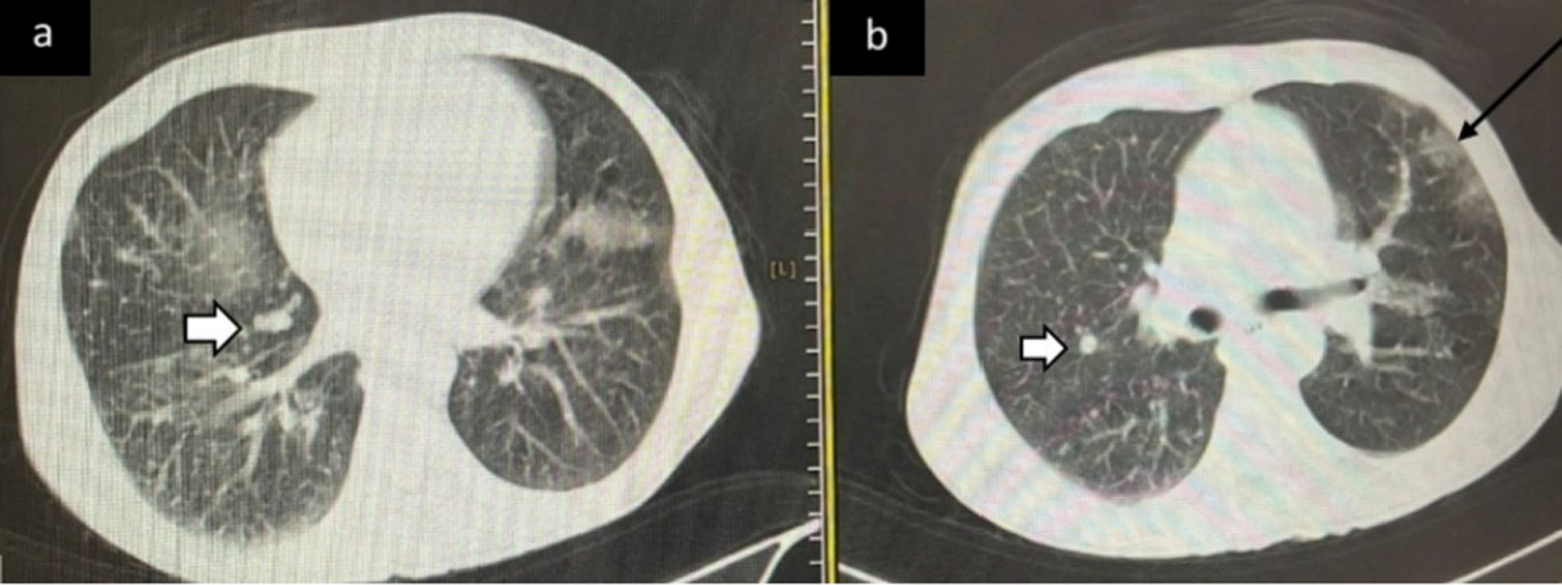
Typical CT imaging features for COVID-19. Unenhanced, axial images of the lungs in a 55-year-old man with hepatocellular carcinoma and positive RT-PCR show peripheral GGO (black arrow, **b**) with metastatic nodules (white arrows, **a**, **b**)

**Fig. 2:**
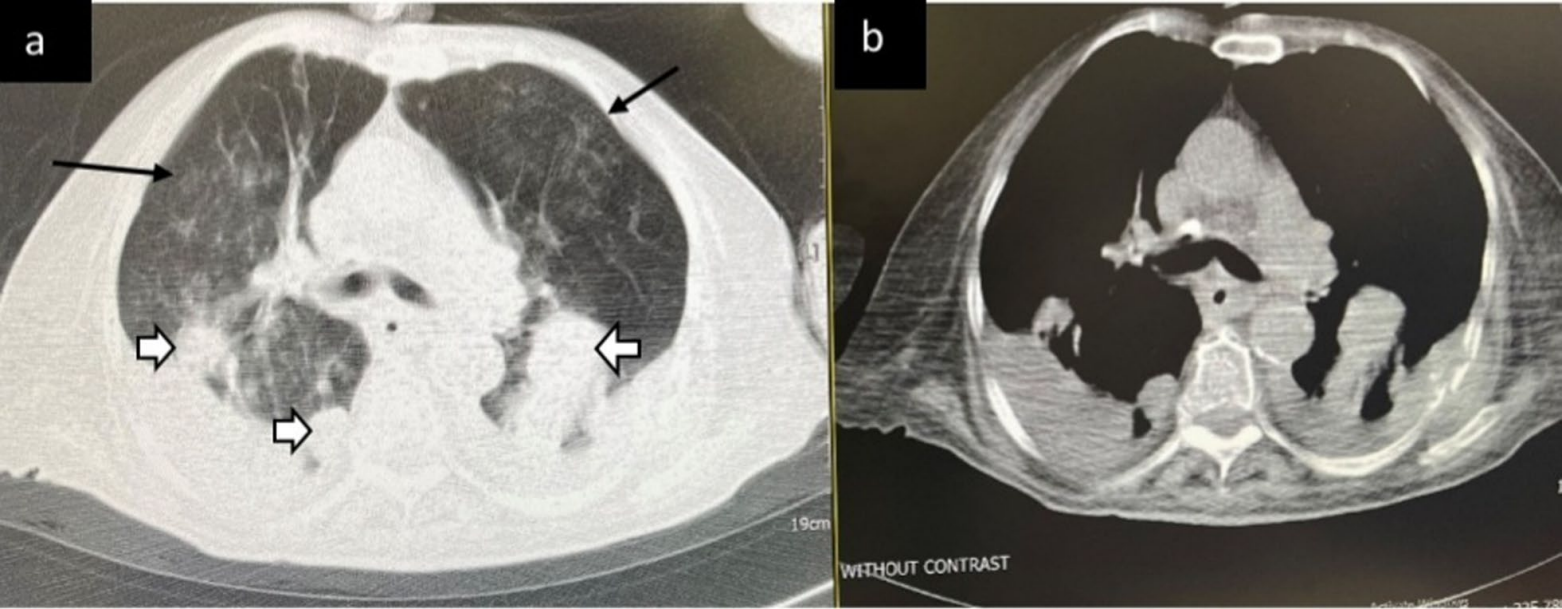
Indeterminate CT imaging features for COVID-19. Unenhanced, axial images of the lung and mediastinal window (**a**, **b**) in an 83-year-old woman with colon cancer and positive RT-PCR shows few, small GGO with a non-rounded and non-peripheral distribution (black arrows) with metastatic nodules and mass (white arrows) and pleural effusion (**b**)

For CT scan involvement scores, all lung lobes were visually evaluated. Each lobe was assigned a score of 0 (non-involvement), 1 (less than 5% involvement), 2 (5–25% involvement), 3 (26–49% involvement), 4 (50–75% involvement), and 5 (> 75% involvement). A total CT score ranging from 0 to 25 was recorded [15]. Furthermore, according to the Radiological Society of North America (RSNA) chest CT classification system for the diagnosis of COVID-19 pneumonia classified CT scans of patients as ‘’Typical’’, ‘’Indeterminate’’, ‘’Atypical’’ and ‘’Negative’’)Fig. 1,2) [9].

### Statistical analysis

We used descriptive statistics to investigate the distribution of the patient’s characteristics. A univariate and multivariate logistic regression model was employed to calculate crude and adjusted odds ratios (OR), 95% confidence interval (CI) and to assess the association between prognostic CT scan factors and COVID-19 mortality. The analysis was repeated for different cancers, and the ORs were adjusted for age, gender, and comorbidities. Stata14 was used for statistical analysis (Stata Statistical Software: Release 14. College Station, TX: Stata Corp LLC).

## Results

The average age of patients was 56.48 (SD: ± 18.59), with 53% male and 47% female. The average hospital stay duration was 13 days (SD: ± 15.2), and the mean oxygen saturation was 90.41 (SD: 8.00). Hypertension (28.2%) and diabetes (25.2%) were the most common comorbidities. Approximately 69% of patients had a positive PCR test, 20.68% had a negative test, and 10.53% had unavailable test results in their hospital records. Thirty-nine percent of cancer patients were admitted to the intensive care unit (ICU), 25.6% required mechanical ventilation, and 33.1% died. The most common type of cancer was GI cancer (n = 78, 29.3%), followed by hematologic (n = 70, 26.3%), breast (n = 28, 10.5%), and lung (n = 14, 5.3%) cancers. The metastatic stage was reported by 78 patients (29.3%). Chemotherapy (n = 174, 65.4%), surgery (n = 115, 43.2%), and radiotherapy (n = 77, 28.9%) were the most commonly used treatments. The mean time elapsed between the last treatment and diagnosis of COVID-19 was 11.02 (SD: ± 39.12) months (Table 1).

**Table 1:** Descriptive analysis of demographic, clinical and laboratory data of all admitted cancer patients with COVID-19 diagnosis

Variable	All cancer 266
Age—Mean (± SD)	56.48 (18.59)
Duration of stay (day)—Mean (± SD)	13.00 (15.20)
Oxygen saturation—Mean (± SD)	90.41(8.00)
Sex	
Female	125 (46.99%)
Male	141 (53.01%)
Comorbidities	
Diabetic	67 (25.19%)
Hypertension	75 (28.20%)
Cardiovascular diseases	50 (18.80%)
Chronic kidney diseases	36 (13.53%)
Lung diseases	31 (11.65%)
Liver diseases	10 (3.76%)
Severity	
ICU admission	104 (39.10%)
Intubation	68 (25.56%)
Dead	88 (33.08%)
Reverse transcriptase-Polymerase Chain Reaction (RT-PCR)	
Negative	55 (20.68%)
Positive	183 (68.80%)
Not done	28 (10.53%)
Type of cancer	
Hematologic**	70 (26.32%)
Lung	14 (5.26%)
Breast	28 (10.53%)
Gastrointestinal	78 (29.32%)
Other	76 (28.57%)
Stage	
Local	11 (4.14%)
Metastatic	78 (29.32%)
Unknown	177 (66.54)
Oncologic treatment	
Surgery	115 (43.23%)
Chemotherapy	174 (65.41%)
Radiotherapy	77 (28.95%)
Target or endocrine therapy	2 (0.75%)
Immunotherapy	1 (0.38%)
Hormonotherapy	11 (4.14%)
Last oncologic treatment interval (Month)	11.02 (± 39.12)

**Hematologic cancer:C42.0, C42.1,C42.4,C77.0, C77.2, C77.4, C77.8, C77.9

We discovered that 42.86% of patients had “typical” imaging features for COVID-19, 21.8% had “atypical”, 21.05% were “indeterminate” for COVID-19, and 14.29% had no finding in the initial chest CT scan (Table 2). “Indeterminate” features were about six times more common than typical features in breast cancer (OR 5.68; 95% CI 1.59–20.21). The involvement score of 18 or higher was 50%, but there was no significant association between the severity score and the cancer type. Among all imaging findings, pleural effusion was significantly prevalent in the lung cancer group (71.43%, *P *value 0.004), and metastatic nodules were more common in the breast cancer group (21.43%, *P *value 0.042) than other findings in these groups.

**Table 2:** Descriptive analysis of chest CT pattern, CT score and classification for COVID-19 of all cancers

	All cancer (N:266,100%)	Hematologic** (N:70,100%)	Lung (N:14,100%)	Breast (N:28, 100%)	Gastrointestinal (N:78,100%)
RSNA classification					
Negative	38 (14.29%)	13 (18.57%)	0	1 (3.57%)	11 (14.10%)
Atypical	58 (21.80%)	10 (14.29%)	5 (35.71%)	8 (28.57%)	17 (21.79%)
Indeterminate	56 (21.05%)	14 (20.00%)	4 (28.57%)	11 (39.29%)	17 (21.79%)
Typical	114 (42.86%)	33 (47.14%)	5 (35.71%)	8 (28.57%)	33 (42.31%)
CT score					
0	95 (35.71%)	23 (32.86%)	5 (35.71%)	9 (32.14%)	28 (35.90%)
≤ 18	39 (14.66%)	13 (18.57%)	1 (7.14%)	7 (25.00%)	9 (11.54%)
> 18	132 (49.62%)	34 (48.57%)	8 (57.14%)	12 (42.86%)	41 (52.56%)
Pattern of involvement					
Normal	95 (35.71%)	23 (32.86%)	5 (35.71%)	9 (32.14%)	28 (35.90%)
Pure GGO	53 (19.57%)	18 (25.71%)	1 (7.14%)	4 (14.29%)	14 (17.95%)
Mixed GGO and consolidation	118 (44.36%)	29 (41.43%)	8 (57.145)	15 (53.57%)	36 (46.15%)
Other abnormality					
Crazy paving	39 (14.66%)	9 (12.865)	3 (21.43%)	5 (17.86%)	11 (14.10%)
Pleural effusion	89 (33.46%)	16 (22.86%)	10 (71.43%)	13 (46.43%)	28 (35.90%)
Pericardial effusion	11 (4.14%)	3 (4.29%)	3 (21.43%)	1 (3.57%)	3 (3.85%)
Lymphadenopathy	33 (12.41%)	10 (14.29%)	2 (14.29%)	3 (10.71%)	6 (7.69%)
Centrilobular nodules	27 (10.15%)	5 (7.14%)	1 (7.14%)	6 (21.43%)	8 (10.26%)
Metastatic nodules	22 (8.27%)	2 (2.86%)	2 (14.29%)	6 (21.43%)	6 (7.69%)
Architectural distortion	22 (8.27%)	4 (5.71%)	2 (14.29%)	3 (10.71%)	8 (10.26%)
Mass	14 (5.26%)	4 (5.71%)	6 (42.86%)	0	0

**Hematologic cancer:C42.0, C42.1,C42.4,C77.0, C77.2, C77.4, C77.8, C77.9

Other cancer: 76 (28.57%) = C00.1, C37.9, C40.2, C40.3, C41.0, C41.2, C41.4, C44.3, C44.5, C44.6, C44.7, C44.9, C48.0, C48.2, C49.2, C49.4, C51.9, C53.9, C54.9, C55.9, C60.9, C64.9, C71.8, C71.9, C72.8, C73.9, C74.9, C76.0, C76.1, C76.2, C76.5, C80.9, C02.9,C03.9, C07.9, C08.9, C09.9, C11.9, C14.0, C31.0, C32.9, C61.9, C67.9

After adjustment for different confounding variables, including age, gender, and comorbidities stratified by cancer type, we discovered that patients with typical CT scan findings in favor of COVID-19 had a roughly threefold higher risk of mortality compared to patients with a normal CT scan (OR 3.47; 95% CI 1.14–8.98) (Table 3). Furthermore, cancer patients with a CT severity score of 18 or higher had a 1.8-fold increased risk of death compared to those with a normal chest CT scan (OR 1.89; 95% CI 1.07–3.34). Consolidation increased mortality risk 1.9 times compared with a normal CT scan, and this risk increased up to 4.5 times when the consolidation was the predominant finding (OR 4.5; 95% CI 1.3–16.2(. Patients with pleural effusion (OR 2.94; 95% CI 1.69–5.12), centrilobular nodule (OR 2.89; 95% CI 1.25–6.65), and architectural distortion (OR 3.76; 95% CI 1.5–9.4) had a worse prognosis compared to patients with a normal chest CT scan.

**Table 3:** Univariate and multivariate analysis of factors associated with outcome, stratified by cancer type*

All cancer				Gastrointestinal		Hematologic	
Variable	Dead/alive	OR1	OR2	Dead/alive	OR2	Dead/alive	OR2
**RSNA classification**							
Negative	6/32	Ref	Ref	5/6	Ref	1/12	Ref
Atypical	20/38	2.80 (1.005–7.83)	2.44 (0.85–6.98)	7/10	0.73 (0.14–3.77)	5/5	11.47 (0.89–147.18)
Indeterminate	17/39	2.32 (0.82–6.58)	2.24 (0.78–6.43)	8/9	1.12 (0.22–5.61)	4/10	4.21 (0.37–47.52)
Typical	45/69	3.47 (1.34–8.98)	**2.99 (1.14–7.86)**	17/16	1.39 (0.31–6.17)	9/24	2.28 (0.21–24.26)
**CT score**							
0	26/69	Ref	Ref	12/16	Ref	6/17	Ref
≤ 18	7/32	0.58 (0.22–1.47)	0.59 (0.23–1.53)	2/7	0.47 (0.07–2.91)	4/9	1.10 (0.22–5.49)
> 18	55/77	1.89 (1.07–3.34)	**1.81 (1.01–3.23)**	23/18	1.94 (0.69–5.45)	9/25	0.53 (0.13–2.16)
**Pattern of involvement**							
Normal	26/69	Ref	Ref	12/16	Ref	6/17	Ref
Pure GGO	14/38	0.99 (0.46–2.12)	0.88 (0 .40–1.93)	4/10	0.48 (0.11–2.17)	4/14	0.51 (0.10–2.55)
Mixed GGO and consolidation	48/70	1.84 (1.03–3.30)	**1.86 (1.03–3.36)**	21/15	2.40 (0.81–7.11)	9/20	0.79 (0.20–3.07)
Predominant GGO	41/65	1.69 (0.93–3.08)	1.68 (0.91–3.09)	19/14	2.34 (0.77–7.06)	8/19	0.70 (0.17–2.86)
Predominant consolidation	7/5	3.76 (1.09–12.9)	**4.55 (1.28–16.17)**	2/1	3.17 (0.23–42.3)	1/1	2.29 (0.11–45.14)
**Other abnormality**							
Crazy paving	16/23	1.49 (0.74–3.00)	1.36 (0.66–2.80)	6/5	1.51 (0.39–5.78)	3/6	0.72 (0.13–3.93)
Pleural effusion	43/46	2.74 (1.60–4.68)	**2.94 (1.69–5.12)**	17/11	2.52 (0.93–6.85)	8/8	**5.34 (1.36–20.90)**
Pericardial effusion	5/6	1.72 (0.51–5.82)	1.75 (0.50–6.06)	1/2	0.93 (0.07–11.52)	1/2	1.20 (0.08–16.27)
Lymphadenopathy	8/25	0.61 (0.26–1.41)	0.58 (0.25–1.37)	2/4	0.54 (0.09–3.29)	3/7	0.92 (0.20–4.28)
Centrilobular nodules	14/13	2.40 (1.07–5.36)	**2.89 (1.25–6.65)**	7/1	**16.88 (1.71–166.38)**	2/3	2.55 (0.32–19.99)
Mass	7/7	2.11 (0.71–6.21)	2.18 (0.72–6.59)	1/0	-	2/2	5.68 (0.54–59.19)
Architectural distortion	13/9	3.25 (1.33–7.94)	**3.76 (1.50–9.40)**	6/2	**7.13 (1.19–42.74)**	3/1	10.53 (0.94–117.32)
Nodular metastatic	10/12	1.77 (0.73–4.28)	1.72 (0.70–4.24)	4/2	2.40 (0.37–15.50)	1/1	5.40 (0.20–140.97)

Bold numbers are indicative of significant OR with P-value < 0.05

*Breast and other cancer not shown in this table because there is no significance in the analysis

OR2 = adjusted by age, gender, comorbidities

COVID-19 mortality had no statistically significant relationship with the crazy paving pattern, pericardial effusion, lymphadenopathy, lung mass, or metastatic nodule.

In GI cancer patients, the risk of mortality was significantly higher for centrilobular nodule (OR 16.9; 95% CI 1.7–166.4) and architectural distortion (OR 7.13; 95% CI 1.19–42.74); and for pleural effusion in hematologic cancer group (OR 5.34; 95% CI 1.36–20.9) in comparison to those who did not have these findings.

## Discussion

In this study, we investigated imaging findings in chest CT of COVID-19 patients with a history of cancer diagnosis. To our knowledge, no comprehensive and well-designed study has yet evaluated chest CT features in patients with malignancy and concurrent COVID-19 infection. The specific imaging findings in this setting are still not fully understood [11–13]. Most publications have merely focused on COVID-19 infection in the general population. A systematic review and meta-analysis published in March 2022 reported on the performance of chest CT for the diagnosis of COVID-19 in patients with and without cancer during the first wave of the COVID-19 pandemic and demonstrated that there is a scarcity of research specifically addressing cancer patients, making evaluating chest CT performance in this population impossible [12].

Our institution’s recently published paper comparing hospital and post-discharge mortality in cancer and non-cancer COVID-19 patients in Iran found that cancer patients have a higher risk of hospital and 60-day mortalities due to COVID-19. Lung cancer patients have the highest risk of COVID-19 death among all cancers. COVID-19 patients with active treatment, metastatic disease, and low SO2 have poor prognoses [16].

The overall case fatality rate in this study was consistent with previously published data on mortality of COVID-19 in cancer patients, ranging from 28 to 31% [7, 17]. Furthermore, our findings support previous research on the correlation between CT scores and disease severity in normal populations [18]. In a study of seventy cancer patients with chest CT evidence of COVID-19, 17 (24%) died after a median follow-up of 25 days. COVID-19 had a median quantitative chest CT extent of 20% in non-survivors and 10% in survivors (*P *value 0.002). COVID-19 pneumonia severity was associated with inpatient management (*P *value 0.003) and oxygen therapy requirements (*P *value < 0.001) [10].

CT features such as crazy-paving patterns, multilobar involvement, consolidations, architectural distortion, and traction bronchiectasis have been linked to a poorer prognosis in normal populations [5, 7–9]. In a study of 28 cancer patients patchy consolidation on CT scans was linked to an increased risk of developing severe events [19]. Our study found similar results and demonstrated that consolidation and architectural distortion play a prognostic role.

In this study, less than half of cancer patients with a COVID-19 diagnosis had typical radiographic features based on the RSNA reporting system. Meanwhile, 42.58% of cancer patients had indeterminate or atypical findings, and 14.29% had normal initial chest CT. Indeterminate results were significantly higher in breast cancer in comparison with other cancers. Furthermore, pattern analysis of chest CT features in different cancers revealed that metastatic nodules are sizably more common in breast cancer. However, pleural effusion is higher in lung cancer. These findings highlight the significance of correlating chest CT results with previous imaging, clinical and epidemiological data, and PCR test results to make the correct diagnosis. Meanwhile, one potential limitation of this study is that CT findings in cancer patients clinically diagnosed with COVID-19 were not compared to their previous chest CT imaging findings because did not have access to previous images of most admitted patients for COVID-19. Future researchers could look into it.

Another potential limitation of our study is including RT-PCR negative cases due to low sensitivity of RT-PCR test at first wave of pandemic, however we analyzed data of positive cancer cases and the result did not change. Although it is not possible to conduct sub analysis for different cancer type due to power issue.

No previous study assessed the various patterns of involvement in cancer patients. More research is needed to evaluate the impact of the RSNA structural report on these specific oncologic settings. A review article published in 2020 by S Katal and colleagues gathered the chest CT scan findings of cancer patients suffering from COVID-19. This study shows that cancer patients with lower immune function may exhibit atypical clinical symptoms or imaging features, such as few or single pulmonary consolidations or GGOs, making the early diagnosis more challenging [11].

As a result, it may be hard to distinguish COVID-19 pneumonia from other non − COVID-19 diseases solely based on chest CT images. Radiologists should be aware that atypical, indeterminate, rare, or subtle CT patterns may be among the presenting radiological features of COVID-19 infection in patients with pre-existing cancer. In the other hand, differential diagnoses of typical ground-glass opacities, such as viral or fungal bronchopneumonia in the immunocompromised state, should be considered. Therefore, in addition to the RT-PCR test, a clinical index of suspicion must be added to CT results to allow for earlier detection and proper treatment of COVID-19, which can be fatal in an oncologic setting.

Our article had the honor of assembling a large sample study of cancer patients diagnosed with COVID-19. For the first time, we investigated the association between the chest CT scan and disease progression and mortality risk.

## Conclusion

In this study, we demonstrated that less than half of cancer patients had typical COVID-19 imaging features. Radiologists should be aware that atypical, rare, or subtle chest CT findings in patients with pre-existing cancer could be COVID-19. There was also a significant correlation between typical COVID-19 imaging features, higher CT scores, the presence of consolidation, pleural effusion, centrilobular nodules, and COVID-19 mortality in cancer patients.

## Data Availability

The datasets used or analyzed during the current study are available from the corresponding author upon reasonable request.

## Abbreviations

COVID-19: Coronavirus disease of 2019
CT: Computerized tomography
GGO: Ground-glass opacity
ICU: Intensive Care Unit
PCR: Polymerase chain reaction
